# Suo Quan Wan Protects Mouse From Early Diabetic Bladder Dysfunction by Mediating Motor Protein Myosin Va and Transporter Protein SLC17A9

**DOI:** 10.3389/fphar.2019.00552

**Published:** 2019-05-24

**Authors:** Jing Wang, Da-wei Lian, Xu-feng Yang, Yi-fei Xu, Fang-jun Chen, Wei-jun Lin, Rui Wang, Li-yao Tang, Wen-kang Ren, Li-jun Fu, Ping Huang, Hong-ying Cao

**Affiliations:** ^1^Department of Pharmacology of Chinese Medicine, School of Pharmaceutical Sciences, Guangzhou University of Chinese Medicine, Guangzhou, China; ^2^Dongguan and Guangzhou University of Chinese Medicine Cooperative Academy of Mathematical Engineering for Chinese Medicine, Guangzhou University of Chinese Medicine, Dongguan, China

**Keywords:** diabetic bladder dysfunction, Suo Quan Wan, overactive bladder, myosin Va, SLC17A9

## Abstract

**Objective:** To investigate the effects of Suo Quan Wan (SQW), a traditional Chinese herbal formula, on the overactive bladder (OAB) of type 2 diabetes mellitus (T2DM) mouse models, particularly on its function of mediating the gene and protein expression levels of myosin Va and SLC17A9.

**Materials and Methods:** After 4 weeks high-fat diet (HFD) feeding, C57BL/6J mice were injected with streptozotocin (100 mg/kg) for four times. After 3 weeks, the diabetic mice were treated with SQW for another 3 weeks. Voided stain on paper assay, fasting blood glucose (FBG) test, and oral glucose tolerance test (OGTT) were conducted. Urodynamic test, tension test [α,β-methylene ATP, electrical-field stimulation (EFS), KCl, and carbachol] and histomorphometry were also performed. Western blot analysis and qPCR assays were used to quantify the expression levels of myosin Va and SLC17A9.

**Results:** The diabetic mice exhibited decreased weight but increased water intake, urine production, FBG, and OGTT. No significant changes were observed after 3 weeks SQW treatment. Urodynamic test indicated that the non-voiding contraction (NVC) frequency, maximum bladder capacity (MBC), residual volume (RV), and bladder compliance (BC) were remarkably increased in the diabetic mice, whereas the voided efficiency (VE) was decreased as a feature of overactivity. Compared with the model mice, SQW treatment significantly improved urodynamic urination with decreased NVC, MBC, RV, and BC, and increased VE. Histomorphometry results showed that the bladder wall of the diabetic mice thickened, and SQW effectively attenuated the pathological alterations. The contract responses of bladder strips to all stimulators were higher in the DSM strips of diabetic mice, whereas SQW treatment markedly decreased the contraction response for all stimuli. Moreover, the protein and gene expression levels of myosin Va and SLC17A9 were up-regulated in the bladders of diabetic mice, but SQW treatment restored such alterations.

**Conclusion:** T2DM mice exhibited the early phase of diabetic bladder dysfunction (DBD) characterized by OAB and bladder dysfunction. SQW can improve the bladder storage and micturition of DBD mice by mediating the protein and gene expression levels of myosin Va and SLC17A9 in the bladder, instead of improving the blood glucose level.

## Introduction

Diabetes mellitus is a common endocrine metabolic disease estimated to affect 629 million individuals in 2045 according to the International Diabetes Federation. T2DM is the extremely prevalent form of diabetes that accounts for 90% of diagnosed cases and is associated with insulin resistance and chronic hyperglycemia (Ferreira et al., 2018). Many clinical studies reported a broad spectrum of lower urinary tract symptoms in diabetic patients (Karoli et al., 2014), accounting for 90–95% of all diabetes cases (Kumar et al., 2013). DBD is a major lower urinary tract complication of diabetes and was first described by Moller (1976). Such complication is traditionally described as a triad of increased capacity, decreased sensation, and poor emptying (Daneshgari et al., 2006) and has affected over 50% of diabetic patients (Dong et al., 2016; Panigrahy et al., 2017). DBD development is divided into two phases: the compensated phase, which occurs in the early phase and is characterized by an OAB; and the decompensated phase, which occurs in the late phase and is characterized by an atonic bladder (Wang et al., 2009; Liu and Daneshgari, 2014).

The pathogenesis of DBD is multifactorial and accompanied by the structural and functional impairments of the bladder (Wang et al., 2017). The bladder structural remodeling of DBD, such as the increase in bladder capacity, total BWT, and smooth muscle content, was observed in STZ-induced diabetic mice. Such remodeling may be a physical alteration to increase the urine volume (Leiria et al., 2011). The two major functions of bladder are urine storage and urine disposal, and the uncoordinated contraction in the OAB of a diabetic greatly affects the urine storage ability of this organ (Daneshgari et al., 2009). Bladder contraction is mainly mediated by purinergic and cholinergic pathways (Liu and Daneshgari, 2005). In particular, ATP is the purinergic messenger released from varicosities or bulbous nerve endings of neurons, and the contractile responses mediated by ATP play a key role in DBD (Yoshizawa et al., 2018). Solute carrier family 17 member 9 (SLC17A9) is a member of the solute-carrier protein family that plays an indispensable role in the vesicular storage of ATP (Sawada et al., 2008; Lazarowski et al., 2011). The translocation of neurotransmitter-filled vesicles to the varicose terminal is the first step in the release of vesicular neurotransmitters, followed by the merger of vesicles with the membrane of the varicose terminal and the precise and rapid release of their contents into the synaptic cleft (Sudhof, 2004). In addition, the motor of vesicles for transportation to the varicose membrane in the cells is mainly provided by myosin motors, particularly myosin Va (Bridgman, 1999). Several studies found that the purinergic inhibitory neurotransmission was impaired in myosin Va-deficient mice (Chaudhury et al., 2012). Such finding suggested that myosin Va played an important role in purinergic neurotransmission (Burnstock, 2014).

Diabetic bladder dysfunction, particularly OAB, is not life threatening to humans. However, this dysfunction seriously affects the quality of the life of patients (Liu and Daneshgari, 2014). The treatment methods for DBD changed when the phase progresses. Anticholinergic drugs, such as tolterodine and solifenacin, are the main treatment options for DBD patients with OAB. However, many side effects, including dry mouth, dry eyes, and memory loss, occurs after the treatment with anticholinergic drugs, thus rendering poor life quality for the patients. In the late phase, surgical intervention was the only therapeutic method for patients who did not benefit from pharmacological and behavioral treatments (Yuan et al., 2015). However, pharmacological and surgical interventions were largely ineffective in clinics (Dong et al., 2016; Wu et al., 2017). Therefore, new effective treatments for DBD are urgently needed.

In the treatment of diabetic OAB, traditional Chinese medicine and natural plant components have recently attracted increasing attention due to their safeness, few side effects, and excellent activity (Xu et al., 2018). SQW is a traditional Chinese herbal formula that was first recorded on *Fu Ren Liang Fang* in the Southern Song Dynasty (between 1127 and 1279 CE). This medicine is a mixture of three Chinese medicines: *roots of Lindera aggregata (Sims) Kosterm. (Lauraceae), roots of Alpinia oxyphylla Miq., (Zingiberaceae), and rhizomes of Dioscorea oppositifolia L. (Dioscoreaceae)* at a 1:1:1 ratio (Hung et al., 2011). SQW has been used to treat lower urinary tract symptoms, such as nocturia, urgency, and child bedwetting for hundreds of years (Cao et al., 2009). We have recently reported that SQW had therapeutic effects on the OAB of bladder outlet obstruction rat models by modulating the TRPV1 expression (Lai et al., 2015). In China, SQW is often used in the clinical treatment of diabetic OAB. However, its mechanism remains unclear, and its therapeutic effect has not been investigated in animal studies. Therefore, we designed experiments to explore the effects and therapeutic mechanisms of SQW in diabetic OAB mouse model.

## Materials and Methods

### Reagents and Materials

Suo Quan Wan was purchased from Hunan Hansen Pharmaceutical Co., Ltd. (China), and the quality control was provided by the company based on Chinese Pharmacopeia employing by high performance liquid chromatography (HPLC) technology from SQW samples (Chinese Pharmacopoeia Commission, 2015). Three Chinese herbals were ground and mixed evenly at a 1:1:1 ratio and appropriate volumes of distilled water were used to make these powders to SQW compound. The doses were adopted according to the Experimental Methodology of Pharmacology, based on clinical usage, the Bios method (Wei et al., 2010). SQW H was 2.208 g/kg, SQW M was 1.104 g/kg, and SQW L was 0.552 g/kg. The tolterodine dose for the positive group was 0.82 mg/kg.

Streptozotocin was purchased from TOKU-E Co., Ltd. (Japan). HFD (45% fat) and control diet were purchased from Guangdong Medical Laboratory Animal Center (China). Tolterodine was purchased from Chengdu Dikang Pharmaceutical Co., Ltd. (China). Roche dynamic Bg meter was purchased from Hoffmann-La Roche Inc. (Switzerland), and carbachol was obtained from Shandong Bausch & Lomb Freda Pharmaceutical Co., Ltd. (China). α,β-methylene ATP was purchased from Tocris Bio-Techne Ltd. (United Kingdom). FastQuant RT Kit (with gDNAse) and Talent qPCR PreMix (SYBR Green) were purchased from TIANGEN Biotech (Beijing) Co., Ltd. (China). TRIzol reagent was purchased from Thermo Fisher Scientific (United States). RIPA lysis buffer and protease inhibitor cocktail (100×) were obtained from CoWin Biosciences (China). All other reagents used were of analytical grade.

### Preparation and HPLC Conditions of SQW

Suo Quan Wan samples were weighted 0.3 g and extracted with 25 mL of methanol-hydrochloric acid solution using heating reflux method and then cool the solution. Finally, the solution was filtered through 0.45 μm nylon membranes before injection.

According to the Chinese pharmacopoeia 2015, the content of norisoboldine should be more than 0.4 mg/0.3 g, and the content of allantoin is more than 0.48 mg/0.3 g. The HPLC conditions and gradient elution were shown as Tables 1, 2.

**Table 1:** Chromatographic condition for norisoboldine.

Column	C18 (25°C)	
Solvent A	Acetonitrile	
Solvent B	0.5% formic acid and 0.1% triethylamine	
Flow rate	1.0 mL/min	
Wavelength	280 nm	

Time (min)	A (%)	B (%)
0	10	90
13	22	78
30	22	78

**Table 2:** Chromatographic condition for allantoin.

Column	C18 (25°C)	
Solvent A	Methanol	
Solvent B	H_2_O	
Flow rate	1.0 mL/min	
Wavelength	191 nm	

Time (min)	A (%)	B (%)
0	8	92
10	10	90
20	10	90

### Animal Model and Treatment

All experimental protocols and animal procedures complied with the ethical principle guidelines of the National Research Council. A total of 100 male C57BL/6J mice (18–22 g) were purchased from Beijing Vital River Laboratory Animal Technology Co., Ltd. and housed in the Experimental Animal Center of Guangzhou University of Chinese Medicine (No. S2017051, Guangzhou, China) under room temperature and exposed to a 12 h/12 h light–dark cycle, with free access to food and water. The animals were fed with normal diet for 3 days and then divided into two groups, namely, diabetic (*n* = 85) and control (*n* = 15) groups. The mice in the diabetic group were fed with HFD, whereas those in the control group received normal diet. After 4-week feeding, the mice in the diabetic group were injected with STZ at 100 mg/kg dissolved in citrate buffer for four times (0.05 M, pH 4.3–4.5). The mice in the control group were treated with an equal volume of vehicle (0.05 M citric acid, pH 4.3–4.5). Fasting Bg (FBG) was measured using an ACCU-CHEK advantage Bg monitoring system (Roche, Indianapolis, IN, United States) through the tail vein 72 h after the last injection. The mice with FBG levels above 11.1 mmol/L were considered diabetic and selected for subsequent experiments. The mice in the diabetic group were divided into five groups: model (*n* = 13), positive (tolterodine, 0.82 mg/kg, *n* = 13), high-dose (SQW H, 2.208 g/kg, *n* = 13), medium-dose (SQW M, 1.104 g/kg, *n* = 13), and low-dose (SQW L, 0.552 g/kg, *n* = 13) groups. After 3-week feeding, the six mouse groups individually received oral gavage of distilled water (control and model group mice), tolterodine, SQW H, SQW M, and SQW L for 3 weeks. During the experiment, the mice in the control group were given normal diet, whereas those in the other groups were continually fed with HFD.

### FBG Test and Oral Glucose Tolerance Test (OGTT)

Fasting blood glucose test and OGTT were conducted after the 3-week SQW treatment. All animals were fasted overnight, and the Bg concentration was measured using a glucometer (ACCU-CHEK active) through a drop of tail blood. All the mice were then given with glucose (2 mg/g body weight) by gavage, and tail blood samples were obtained at 0, 15, 30, 60, 90, and 120 min to measure the Bg concentration. The area under the curve of the Bg time course from 0 to 120 min (AUC_0-2__h_) was calculated according to the following formula:

$$\begin{array}{c} {\text{AUC}}_{0-2\text{h}}=[(\text{Bg}0+\text{Bg}15)]\times 0.5]÷4+[(\text{Bg}15+\text{Bg}30)\times 0.5]÷4\\ +[(\text{Bg}30+\text{Bg}60)\times 0.5]÷2+[(\text{Bg}60+\text{Bg}120)\times 0.5] \end{array}$$

### Measurement of Water Intake, Urine Output, and Frequency

The mice were placed individually in metabolic cages for 24 h with food and water *ad libitum*. Water intake was measured based on the water consumption for 24 h. Urine output and micturition frequency were analyzed through the VSOP test. Urine output was measured by evaluating the volume of urine in the collector after the mice were placed in the cages for 5 h. Micturition frequency was measured by visualizing and analyzing the collected papers, which were placed under the metabolic cage for 5 h under ultraviolet light to identify the area of urine spots (Wang et al., 2012). The sizes of the urine spots were divided into two levels, namely, bigger volume (>50 μL) and smaller volume (<50 μL) voids, to measure the micturition frequency.

### Urodynamic Test

The urodynamic test was performed using a micro-injection pump and urodynamic measuring device (Laborite Delphis 94-R01-BT, Canada). All mice were anesthetized by the intraperitoneal injection of 25% urethane (2.0 mg/kg). A ventral midline incision was made to expose the bladder, and a 25-gauge needle was inserted into the bladder dome and fixed with silk suture. The needle was connected through a three-way adapter, which was connected with urodynamics at one end and a micro-injection pump at the other. After the bladder was emptied, 0.9% saline solution was injected into the bladder through the micro-injection pump at a rate of 3 mL/h. Pumping was stopped immediately when urine was observed at the external urethra. The bladder pressure line was automatically recorded with a computer. Urodynamic test parameters included the frequency of NVC (higher than 4 cm H_2_O spontaneous bladder contraction that did not result in urination before first urination) frequency, MBC (the volume of saline pumped before first urination), maximum voiding pressure (MP, the maximum peak pressure reached during micturition), RV (manually drained and measured with 1 mL syringe), MV (calculated as MBC - RV), VE (calculated as [(MBC - RV)/MBC] × 100%), and BC [calculated as (MBC/MP) × 100%]. The mice were euthanized at the end of the experiment through cervical dislocation (Lai et al., 2016).

### Histological Test

After the mice were euthanized, the bladders were excised and fixed using 4% paraformaldehyde solution for approximately 24 h at room temperature. After fixation, the bladders were conventionally dehydrated and embedded in paraffin. The tissues were cut into 6 μm thickness and stained with hematoxylin and eosin (HE) and Masson’s trichrome. The color segmentation of Masson’s trichrome was used to identify the whole cross-sectional area and the tissue areas that were stained “pink” (urothelium), “blue” (collagen), and “red” (smooth muscle). The HE images (100×) were used to determine the BWT, whereas the Masson’s trichrome stained images (100×) to measure the smooth-muscle-to-collagen ratio. The stained bladder sections were examined under a light microscope, and representative images were photographed with a digital camera mounted on the microscope. All the images were analyzed using image analysis software (Image Pro 6.0).

### Assessment of Detrusor Smooth Muscle Contractility Study *in vitro*

The mice were executed, and the bladders were excised at the bladder neck. Full-thickness longitudinal DSM strips (0.7–1 mm × 5 mm) were obtained and mounted in a 5 mL organ bath filled with Krebs-Henseleit solution (NaCl, 118 mM; KCl, 4.75 mM; MgSO_4_, 1.18 mM; NaHCO_3_, 24.8 mM; KH_2_PO_3_, 1.18 mM; CaCl_2_ 2.5 mM; and C_6_H_12_O_6_⋅H_2_O, 10 mM; pH = 7.4) bubbled with a mixture of 5% carbon dioxide and 95% oxygen at 37°C. One side of the strip was attached to the hook with silk suture, and the other side was connected to the force signal transducer (Wang et al., 2012). The passive tension was loaded at 0.5 g, and the tissues were equilibrated for 60 min before the experiments. The forced change signals of the DSM strips were recorded with a PowerLab recorder. Purinergic agonist, α,β-methylene ATP (100 μM) was added to the organ bath twice (30 min between each assay) to measure the difference in the contractile responses. The contraction of bladder tissue to electrical field stimulation (EFS, 1, 2, 4, 8, 16, 32, and 64 Hz; 40 V; and 0.5 ms pulse duration for 10 s) was also measured. Furthermore, tests for dose–response curve to carbachol (10^-8^–10^-5^ M) and the contractile response to KCl (120 mM) were performed in the DMS strips. At the end of the experiments, the weight and length of each detrusor strip were recorded.

### Real-Time RT-PCR

The total RNA from the whole bladder samples were extracted using TRIzol Reagent (Invitrogen, United States). The absorption of the samples at 260 and 280 nm was used to estimate the RNA quality. A260/A280 was used to check the purity, and A260 values confirmed the concentration of RNA (Shimadzu BioSpec-nano, Japan). The total RNA was reverse transcribed into cDNA using a PrimeScript RT Reagent Kit with gDNA eraser (TIANGEN, China). Real-time PCR analysis was performed using SYBR Green (TIANGEN, China) according to the manufacturer’s instructions. Synthetic oligonucleotide primers were designed to amplify the cDNA for the genes encoding the myosin Va, SLC17A9, and β-actin. Table 3 shows the primer pairs. The reaction program was presented as follows: 95°C for 3 min, followed by 39 cycles at 95°C for 5 s and 55°C for 10 s. Results were recorded and analyzed using complementary software, and the gene expression levels were calculated by 2^-ΔΔCt^ method. The target gene expression levels were individually normalized according to the β-actin expression.

**Table 3:** Primer sequences of myosin Va, SLC17A9, and β-actin.

Gene	Primers (5′–3′)
*myosin Va - F*	AGCTCAACTCCTTCCACTC
*myosin Va - R*	ACACACCCCTTTATCCTTCC
*SLC17A9 - F*	GCTTCCTCAAGGCTATGATCTT
*SLC17A9 - R*	AGGTCCTGAATGTTGACTGAAA
*β-actin - F*	CTACCTCATGAAGATCCTGACC
*β-actin - R*	CACAGCTTCTCTTTGATGTCAC

### Western Blot Analysis

The bladder tissues were homogenized using tissue grinders (Shanghai Jingxin, Shanghai, China) at 65 Hz for 2 min to extract the total protein. BCA protein assay Kit (Beyotime Biotechnology, China) was used to measure the protein concentration. Equivalent proteins (20 μg) were subjected to 10% or 8% SDS-PAGE at 80 V for 30 min or 120 V for 60 min, respectively, to separate the proteins of different molecular weights and transfer to the PVDF membranes using the transblotting apparatus (Bio-Rad Laboratories, Hercules, CA, United States) for 55 or 110 min, respectively, at 300 mA. The PVDF membranes were blocked with 5% (w/v) non-fat milk buffer at room temperature for 2 h and incubated with a primary antibody in TBST [Myosin Va (1:1000, Santa Cruz), SLC17A9 (1:1000, MBL), or β-actin (1:1000, 4A Biotech)] overnight at 4°C. The immune-labeled membranes were washed three times with TBST for 15 min each time, and then conjugated with a secondary antibody (1:5000, 4A Biotech) at room temperature for 2 h. After the non-binding secondary antibodies were washed away, the target protein bands were visualized using a chemiluminescent reagent (Millipore, United States). Data were processed using ImageJ, and the immunoblot protein expression levels of myosin Va and SLC17A9 were normalized using β-actin. The antibodies used in the present study are listed in Supplementary Table 1.

### Statistical Analysis

All data were expressed as mean ± SD. Statistical analyses were performed using SPSS 19.0 (SPSS, United States). The amplitude of contractile responses to stimulus was recorded in tension (Newton) and normalized by the weight (g) of the detrusor strips (Chaudhury et al., 2014). Western blot analysis data were processed using ImageJ. Histological test images were analyzed using Image-Pro Plus 6.0, and one-way ANOVA was used for data analysis. *P* < 0.05 was considered statistically significant.

## Results

### HPLC Analysis of SQW

**FIGURE 1:**
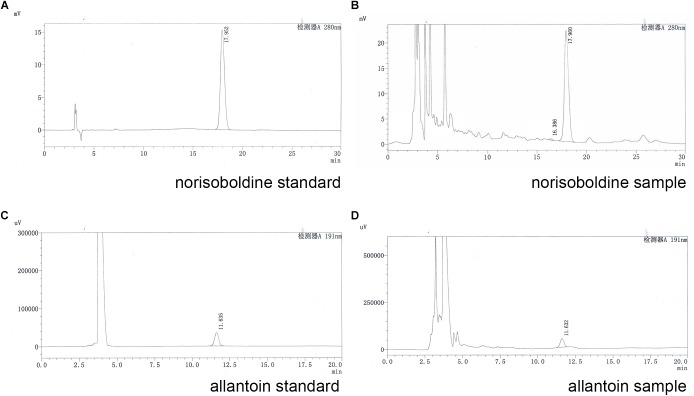
HPLC chromatogram of standards and samples; **(A)** norisoboldine standard; **(B)** norisoboldine sample; **(C)** allantoin standard; **(D)** allantoin sample.

For quality assessment of SQW, HPLC analysis was conducted. The detection wavelength of norisoboldine was set at 280 nm and the allantoin was set at 191 nm. The retention times of norisoboldine and allantoin were detected at approximately 17.960 and 11.632 min, respectively (Figure 1A–D). According to the chromatograms results, the contents of norisoboldine and allantoin in SQW sample were 0.72 mg/0.3 g and 0.73 mg/0.3 g, respectively, indicating that the SQW samples meet the requirement.

### General Characteristics of the Diabetic Model

**FIGURE 2:**
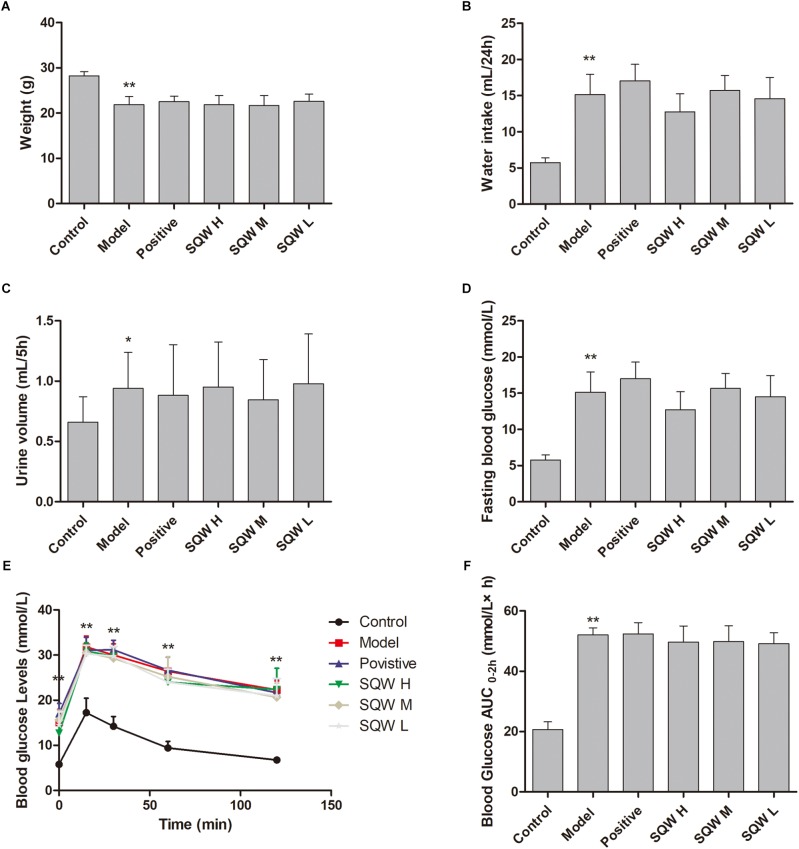
Effects of SQW on the general characteristics of diabetic model after the 3-week treatment (*n* = 8). **(A)** Weight, **(B)** water intake, **(C)** 5 h urine volume, **(D)** FBG, **(E)** OGTT, and **(F)** area under the curve of AUC_0-2__h_ (calculated according to the following formula: AUC_0-2h_ = [(Bg0 + Bg15) × 0.5] ÷ 4+ [(Bg15 + Bg30) × 0.5] ÷ 4+[(Bg30 + Bg60) × 0.5] ÷ 2+[(Bg60 + Bg120) × 0.5]). Data represent the means ± SD (model vs. control group, ^∗∗^*P* < 0.01 or ^∗^*P* < 0.05).

Compared with the mice in the control group, the T2DM mice exhibited diabetes characteristics, including significantly reduced weight (*P* < 0.01) and increased water intake (*P* < 0.01), urine volume (*P* < 0.01), Bg levels [high FBG (*P* < 0.01), OGTT (*P* < 0.01), and AUC_0-2h_ (*P* < 0.01)]. No considerable differences in these parameters were observed among the mice in SQW and model groups (Figure 2A–F).

### VSOP and Urodynamic Tests

**FIGURE 3:**
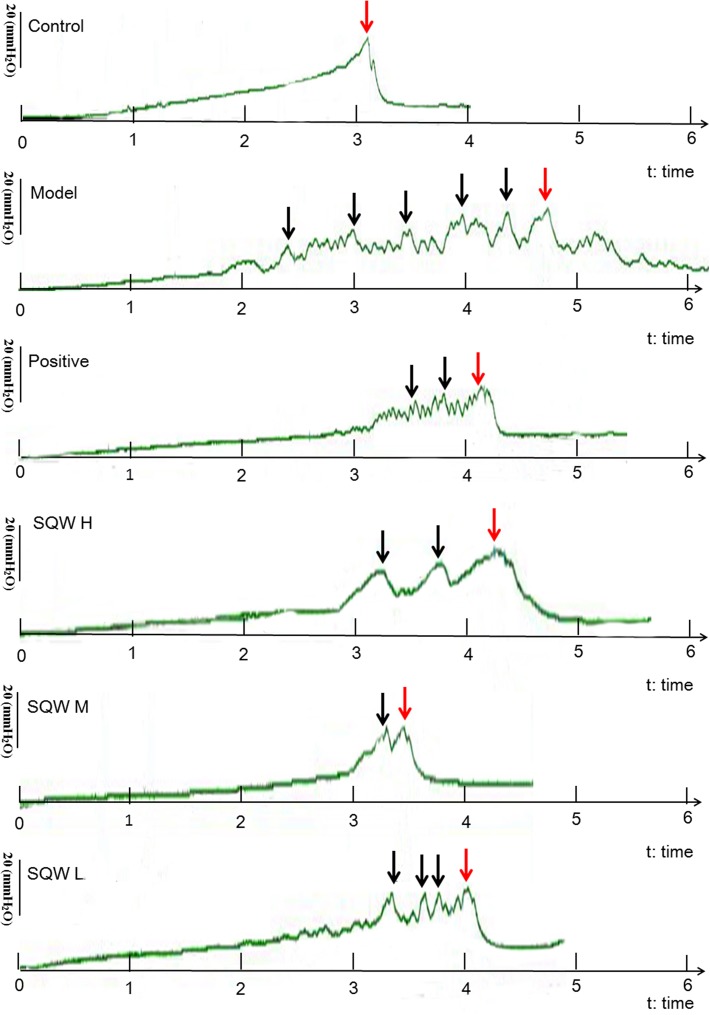
Representative urodynamic test recording from the six groups of mice. Red arrows indicate the micturition peaks, and black arrows represent the NVC frequency.

**FIGURE 4:**
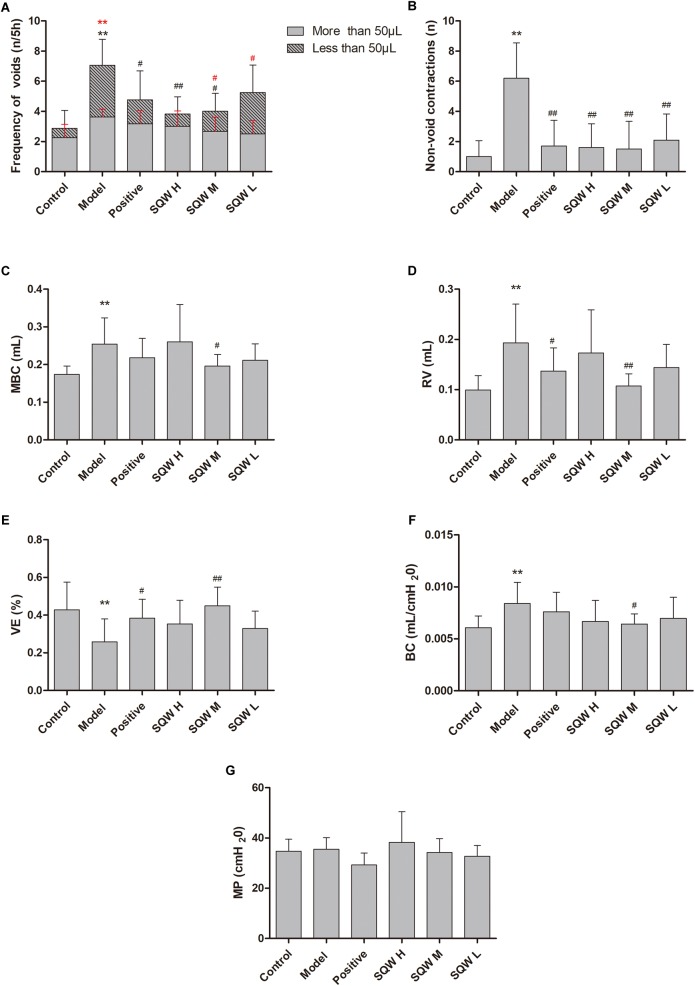
VSOP and urodynamic test results in all groups (*n* = 8). **(A)** Frequencies of bigger volume (>50 μL) and smaller volume voids (<50 μL); **(B)** Frequency of NVC before the first micturition; **(C)** MBC; **(D)** RV; **(E)** VE; **(F)** BC; and **(G)** MP. Data represent the means ± SD (model vs. control group, ^∗∗^*P* < 0.01; treatment vs. model group ^#^*P* < 0.05 or ^##^*P* < 0.01).

The representative urodynamic response curves of each group are presented in Figure 3. The urinary voiding patterns showed that the frequencies of bigger volume voids (>50 μL) and smaller volume voids (<50 μL) were higher in diabetic mice than in the controls Figure 4A. Treatment with SQW M markedly decreased both frequencies (*P* < 0.05), whereas treatments with SQW H and SQW L reduced the frequencies of smaller volume and bigger volume voids, respectively. In addition, urodynamic test revealed that compared with the controls, the diabetic mice had significantly increased NVC, MBC, RV, and BC (*P* < 0.01) but markedly decreased VE (*P* < 0.01), thereby showing typical DBD in the early compensated phase (Figure 4B–F). SQW M treatment remarkably decreased the NVC, MBC, RV, and BC (*P* < 0.01 or *P* < 0.05) but significantly increased the VE of the mice (*P* < 0.01). Furthermore, treatments with SQW H and SQW L remarkably decreased the NVC of the mice (*P* < 0.05). No significant differences in MP were found among the control, SQW-treated, and model mice (Figure 4G).

### Morphometric Analysis

**FIGURE 5:**
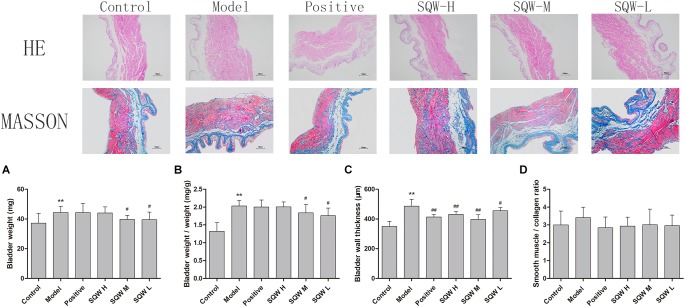
Bladder weight and digital images (100×) of HE and Masson’s trichrome staining from the six groups of mice (*n* = 8). **(A)** Bladder weight; **(B)** bladder-weight-to-body-weight ratio; **(C)** BWT measured from HE images; **(D)** smooth-muscle-to-collagen ratio determined by the Masson’s trichrome images. Data represent the means ± SD (model vs. control group, ^∗∗^*P* < 0.01; treatment vs. model group ^#^*P* < 0.05 and ^##^*P* < 0.01).

The bladder weight (absolute and relative to body weight) was increased in the diabetic mice (*P* < 0.01) but decreased in the SQW M- and SQW L-treated mice (*P* < 0.05) compared with that in the controls (Figure 5A,B). The results of the morphometric analysis were consistent with the bladder weight. The BWT was significantly increased in diabetic mice (*P* < 0.01), but SQW treatment effectively inhibited this alteration (Figure 5C). No substantial differences in the smooth-muscle-to-collagen ratio were observed among the control, SQW-treated, and model mice (Figure 5D).

### Contractility Studies *in vitro*

**FIGURE 6:**
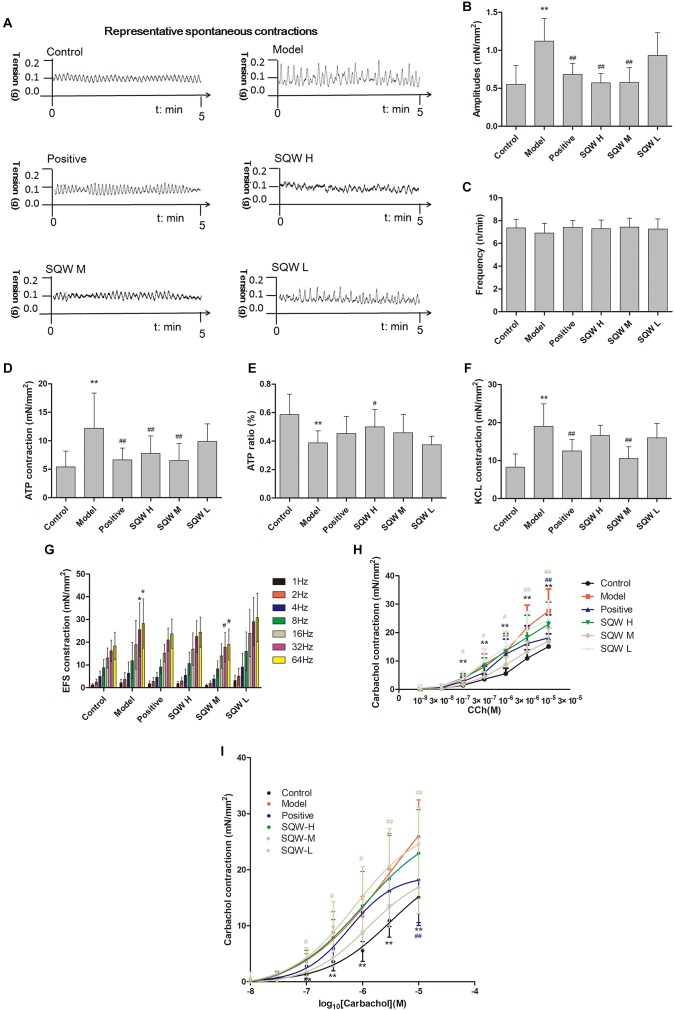
DSM strips of diabetic mice exhibited high amplitudes of spontaneous activity and increased bladder contractions to stimuli, and SQW treatment inhibited the changes (*n* = 5). **(A)** Representative spontaneous contractions of the bladder detrusor strips from the mice in the six groups. Quantification of the **(B)** amplitude and **(C)** frequency of spontaneous contraction; **(D)** DSM strip contractions induced by α,β-methylene ATP (100 μM). **(E)** ATP ratio (calculated as [before-cons – after-cons]/pro-cons), α,β-methylene ATP (100 μM)-induced contractions (two times added α,β-methylene ATP); **(F)** DSM strip contractions induced by KCl (120 mmol/L); and **(G)** DSM strip contractions induced by EFS (1–64 Hz); and **(H)** carbachol (10^-8^–10^-5^ M); **(I)** The cumulative concentration–response curves of carbachol. Data represent the means ± SD (model vs. control group, ^∗^*P* < 0.05 or ^∗∗^*P* < 0.01; treatment vs. model group, ^#^*P* < 0.05 or ^##^*P* < 0.01).

We found that the DMS strips of diabetic mice exhibited significantly higher amplitudes of spontaneous activity that those of the controls (*P* < 0.01). SQW H and SQW M treatments markedly decreased this alteration (*P* < 0.01) (Figure 6A–C). α,β-methylene ATP (100 μM), which is the P2X receptor agonist, caused higher contractions in the DMS strips of diabetic mice compared with those of the controls (*P* < 0.01). α,β-methylene ATP (100 μM) was added twice to activate the bladder strip. The responses evidently increased in the first reaction but markedly decreased in the second response in the diabetic mice compared with those in the controls (*P* < 0.05). SQW H treatment markedly reverted all these alterations (*P* < 0.01 or *P* < 0.05), and SQW M treatment decreased the ATP-induced contractions (Figure 6D,E). In addition, the contractions caused by KCl (120 mM), EFS (1–64 Hz) were higher in the diabetic mice than in the controls (*P* < 0.01 or *P* < 0.05). The cumulative concentration -response curve of carbachol (10^-8^–10^-5^ M) was also higher in the diabetic mice than in the controls (*P* < 0.01 or *P* < 0.05). The contractions of the DSM strips were markedly decreased due to the treatment with SQW (*P* < 0.01 or *P* < 0.05) (Figure 6F–I). No significant differences in pEC50 were found among the control, SQW-treated, and model mice. The Emax of diabetic mice exhibited significantly higher than the controls (*P* < 0.01). Positive and SQW-M treatments markedly decreased (*P* < 0.01) (Table 4).

**Table 4:** The pEC50 and Emax of carbachol (means ± SD, *n* = 5).

Group	Dose	pEC50	Emax
Control	–	5.68 ± 0.38	15.07 ± 4.51
Model	–	5.93 ± 0.20	27.58 ± 7.76^∗∗^
Positive	0.82 mg/kg	6.23 ± 0.21	18.14 ± 8.11^##^
SQW-H	2.208 g/kg	5.97 ± 0.65	22.95 ± 7.78
SQW-M	1.104 g/kg	5.94 ± 0.32	16.86 ± 4.68^##^
SQW-L	0.552 g/kg	6.15 ± 0.24	24.59 ± 6.60

*Model vs. control group, ^∗∗^P < 0.01; treatment vs. model group ^##^P < 0.01.*

### Real-Time RT-PCR Analysis

**FIGURE 7:**
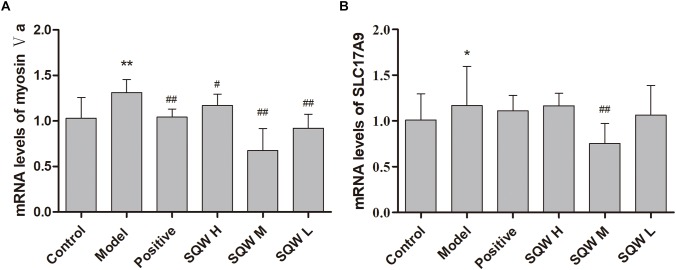
Effects of SQW treatment on the mRNA expression levels of myosin Va and SLC17A9 in the bladder tissues (*n* = 8). Quantification of mRNA expression levels of **(A)** myosin Va and **(B)** SLC17A9 normalized with β-actin by 2^-ΔΔCt^ method. Data represent the means ± SD (model vs. control group ^∗^*P* < 0.05 or ^∗∗^*P* < 0.01; treatment vs. model group ^#^*P* < 0.05 and ^##^*P* < 0.01).

According to the results of RT-PCR analysis, the mRNA expression levels of myosin Va and SLC17A9 were significantly increased in the diabetic mice compared with those in the control mice (*P* < 0.01 or *P* < 0.05). The 3-week SQW M treatment, markedly decreased the mRNA expression levels of myosin Va and SLC17A9 (*P* < 0.01 or *P* < 0.05), whereas the SQW H and SQW L treatments reduced the myosin Va mRNA expression level only (*P* < 0.01 or *P* < 0.05) (Figure 7A,B).

### Western Blot Analysis

**FIGURE 8:**
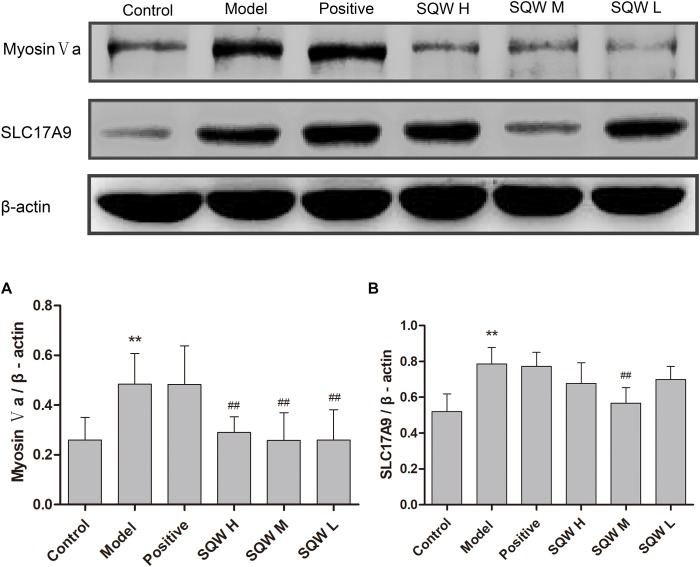
Representative immunoblots of the protein expression levels of myosin Va, SLC17A9; and β-actin and effects of SQW treatment on these expression levels in the bladder tissues (*n* = 8). Quantification of protein expression of **(A)** myosin Va and **(B)** SLC17A9 by normalizing with β-actin. Data represent the means ± SD (model vs. control group, ^∗∗^*P* < 0.01; treatment vs. model group, ^##^*P* < 0.01).

The protein expression levels of myosin Va, SLC17A9, and β-actin were evaluated through Western blotting. The results showed that the protein expression levels of myosin Va and SLC17A9 were significantly increased in the bladder tissues of diabetic mice compared with those in the controls (*P* < 0.01). After the 3-week SQW treatment, SQW M treatment markedly decreased the protein expression levels of myosin Va and SLC17A9, whereas SQW H and SQW L treatments significantly reduced the protein expression of myosin Va only (*P* < 0.01) (Figure 8A,B).

## Discussion

Diabetic bladder dysfunction is a major lower urinary tract complication of diabetes, but its molecular mechanism remains unknown. Several studies reported that rat models with STZ-induced T2DM usually exhibited major clinical urodynamic alterations (Daneshgari et al., 2006; Leiria et al., 2011; Wang et al., 2012). We have previously reported that TCM, namely SQW, had therapeutic effects on many lower urinary tract diseases, including operation-induced OAB and outlet obstruction (Lai et al., 2015). Two components (norisoboldine and allantoin) were detected as quality standards in SQW, and the results of HPLC showed that the active ingredient content met the standard. SQW is an oral traditional Chinese formula for the treatment of lower urinary tract diseases, and the oral dose is 5.4 g/day. The high dose in our study was double the clinical dose, and the middle dose was equation, while the low dose was half. Also, we calculated the equivalent doses of mice administration according to the equation (Wei et al., 2010). In the present study, we explored the effects and potential mechanism of SQW on diabetic OAB by using STZ-induced T2DM mouse model.

The results of our research showed that the model mice were characterized by a range of T2DM symptoms, including abnormal fat, carbohydrate metabolism, and high Bg levels, thereby indicating the successful establishment of the T2DM model. Our VSOP and urodynamic tests showed significant alternations in the micturition of diabetic mice as characterized by increased frequency of voids (smaller micturition was apparent), NVCs, MBC, BC, and RV and markedly reduced VE after 6 weeks of hyperglycemia status. These results indicated that the diabetic mice entered the stage of OAB, which is consistent with those of previous studies (Leiria et al., 2011; Wang et al., 2012). SQW treatment effectively improved the bladder function of the T2DM mice but did not change the high Bg status.

In healthy bladders, the contractions caused by ATP were limited (Leiria et al., 2011). However, in the early phase of DBD, the purinergic-induced contractions account for up to 150% compared with those in healthy individuals (Bayliss et al., 1999). The transfer of ATP-filled vesicles to the varicose membranes and the effects of DBD on the transfer mechanism were poorly explored. Recent studies have provided key information about the contributions of SLC17A9 and myosin Va in the storage and exocytosis of ATP in secretory vesicles (Sawada et al., 2008; Chaudhury et al., 2012). SLC17A9 is a vesicular nucleotide transporter that plays an essential role in the specific transport of ATP inside purinergic vesicles (Rudnick, 2008). This transporter was discovered in various invertebrates and vertebrates, indicating that the molecular mechanisms of purinergic transmission are common in animals (Sawada et al., 2008). Accurate localization of the neurotransmitter-filled vesicles in varicose membranes is indispensable for exocytosis (Rizzoli and Betz, 2005). The local transport of organelles, including purinergic vesicles, requires energy that is provided by molecular motors, such as myosin Va. Myosin Va is a subtype of the unconventional myosin V and is primarily expressed in the central and peripheral nervous systems and melanocytes (Trybus, 2008). The structure of myosin Va is provided for the continuous forward transport of intracellular cargoes (Skyrmejones et al., 2000), and its movement changes with cargo binding (Ikebe and Ikebe, 2008). Recent studies reported that myosin Va played a key role in purinergic vesicular transport and was closely associated with ATP-containing vesicles by binding directly to SLC17A9 (Chaudhury et al., 2012). These findings provided us the research direction to explore the effects of SQW and the alterations in purinergic vesicular transport in DBD mice.

The results *in vitro* contractility study on the bladder were in accordance with the “temporal theory” of DBD. In the early phase, the DSM of diabetic mice exhibited markedly high amplitudes of spontaneous activity and increased responsiveness to stimuli, such as α,β-methylene ATP, KCL, EFS, and carbachol (Daneshgari et al., 2006). *In vitro* studies the results showed that the DMS of diabetic mice exhibited markedly high amplitudes of spontaneous activity compared with those of the controls, and the waves were disordered. The frequency of spontaneous activity remained stable, which was consistent with a previous report (Wang et al., 2012). Compared with those of the controls, the contractile responses of the DSM strips of diabetic mice to α,β-methylene ATP were substantially increased during the first response but markedly decreased during the second time. These results suggested that the DSM strips of diabetics exhibited increased responsiveness to exogenous purinergic agonists, and the bladder remained high responsiveness after the first stimulate in diabetic mice. In agreement with the above findings, the contractions of DSM strips to KCL, EFS, and carbachol were high in the diabetic mice. After the 3-week SQW treatment, the mice in the SQW treatment groups displayed varying degrees of reduction for their contractile responses to all stimulus, especially to α,β-methylene ATP, as compared with the models. This result was consistent with those of VSOP and urodynamic test, thus further confirming the therapeutic effects of SQW in the OAB of diabetic mice.

The hyper-responsiveness of diabetic DSM to α,β-methylene ATP, KCL, EFS, and carbachol reflects the changes at the neurotransmitter level and/or beyond the neurotransmitters related to the alterations in the upstream vesicular nucleotide transporters. Therefore, we evaluated the expression levels of protein and mRNA for myosin Va and SLC17A9. In the bladders of the models, we found high expression levels of protein and mRNA for myosin Va and SLC17A9, indicating that the increased expression of these genes is the potential pathogenic mechanism of diabetic OAB. Moreover, we found that the expression of levels of proteins and mRNA of myosin Va and SLC17A9 were significantly decreased after SQW treatment, suggesting that the downregulation of expression levels of myosin Va and SLC17A9 contributes to the therapeutic effect of SQW in DBD.

In this study, SQW was demonstrated to have effective treatment on DBD, and the possible mechanisms were also explored. However, the active ingredients of SQW are unclear, since the complexity of its component. To further explore the major effective ingredient in SQW is our goal in the future.

We want to explore weather a single herbal has effects in DBD. In the present study, we explored the effects and potential mechanism of SQW on diabetic OAB by using STZ-induced T2DM mouse model. SQW is one of the most commonly traditional Chinese formula to treat various urinary system diseases in China for thousands of years (Lai et al., 2015), such as urinary incontinence, nocturnal enuresis and OAB symptom syndrome (Lai et al., 2016; Lee et al., 2018). And diabetic OAB has the same symptom like nocturia and urgency, SQW also was used to treat DBD or combined with other drugs in clinical (Liu and Lan, 2006; Chen et al., 2018). Studies has already found some mechanisms, such as β receptor, P2X, TRPV1 (Lai et al., 2016; Xu et al., 2017), but its complicated. We intend to conduct further research, learn its potential herbal components. Study found that radix linderae extracts have effects on OAB and diabetic bladder (Tan et al., 2016; Yang et al., 2019). The main components in radix linderae is norisoboldine and ursolide (Chen et al., 2013; Na et al., 2017). We intend to conduct further research on efficacy and mechanism using the component in radix linderae.

## Conclusion

In summary, our study revealed that HFD with STZ-induced T2DM model mice showed OAB symptoms after 6 weeks of hyperglycemia status. In addition, the traditional Chinese formula, SQW, exhibited therapeutic effects on the OAB of model mice. SQW directly targeted the bladder, rather than improving the Bg levels. The mechanism was related to the inhibition of the transmission of purinergic neurotransmitters in the bladder of diabetic mice by downregulating the expression levels of myosin Va and SLC17A9.

## Ethics Statement

This study was carried out in accordance with the ethical principle guidelines of the National Research Council. The experimental protocol was approved by the Committee on Ethics of Guangzhou University of Chinese Medicine.

## Author Contributions

H-yC conceived and designed the study. PH directed the experiments. JW, X-fY, W-jL, RW, L-yT, W-kR, L-jF, F-jC, and D-wL constructed the animal model. JW, X-fY, and Y-fX analyzed the data and drafted the manuscript. D-wL made a great contribution in the revision of the later articles. All authors read and approved the final manuscript.

## Conflict of Interest Statement

The authors declare that the research was conducted in the absence of any commercial or financial relationships that could be construed as a potential conflict of interest.

## Abbreviations

ANOVA: analysis of variance
BC: bladder compliance
Bg: blood glucose
BWT: bladder wall thickness
DBD: diabetic bladder dysfunction
DSM: detrusor smooth muscle
EFS: electrical-field stimulation
FBG: fasting blood glucose
HFD: high-fat diet
MBC: maximum bladder capacity
MV: micturition volume
NVC: non-voiding contraction
OAB: overactive bladder
OGTT: oral glucose tolerance test
RV: residual volume
SD: standard deviation
SQW: Suo Quan Wan
SQW H: high-dose SQW
SQW L: low-dose SQW
SQW M: medium-dose SQW
STZ: streptozotocin
T2DM: type 2 diabetes mellitus
VSOP: voided stain on paper
VE: voided efficiency
