# Supplementary material for: Suo Quan Wan Protects Mouse From Early Diabetic Bladder Dysfunction by Mediating Motor Protein Myosin Va and Transporter Protein SLC17A9
Source: Front Pharmacol. 2019 May 24;10:552. doi: 10.3389/fphar.2019.00552 (PMC6543251; doi:10.3389/fphar.2019.00552)
Supplement: Supplementary file 1 [file Data_Sheet_1.PDF]

Supplementary Material

Suo Quan Wan protects mouse from early diabetic bladder dysfunction by mediating motor protein myosin Va and transporter protein SLC17A9

Jing Wang[†], Da-wei Lian[†], Xu-feng Yang, Yi-fei Xu, Fang-jun Chen, Wei-jun Lin, Rui Wang, Li-yao Tang, Wen-kang Ren, Li-jun Fu, Ping Huang, Hong-ying Cao*

†These authors have contributed equally to this work

*** Correspondence:** Hong-ying Cao: 1171629708@qq.com

Suo Quan Wan (SQW) was purchased from Hansen Pharm (Batch No. 1707201). The doses for adults were 6 grains one time and three times every day (0.3 g per grain). The quality control of SQW strictly followed the Chinese pharmacopoeia 2015 based on high performance liquid chromatography (HPLC) technology (Commission, 2015), the two herbs asked for detection were *Lindera aggregata (Sims) Kosterm., fructus, and Dioscorea opposita Thunb. Rhizome.* The two dominating compounds were norisoboldine and allantoin, and the data were provided by Hansen Pharm.

1. Preparation of SQW

SQW is a traditional Chinese herbal formula that was first recorded on Fu Ren Liang Fang. This medicine is a mixture of three Chinese medicines: *roots of Lindera aggregata (Sims) Kosterm. (Lauraceae), roots of Alpinia oxyphylla Miq., (Zingiberaceae), and rhizomes of Dioscorea oppositifolia L. (Dioscoreaceae)* at a 1:1:1 ratio and appropriate volumes of distilled water were used to make these powders to SQW compound.

2. The dosage of Suo Quan Wan (SQW) in mice

The dosage of SQW in mice was calculated by the oral dose of human in clinical, according to Experimental Methodology of Pharmacology, based on clinical usage, the Bios method(Wei et al., 2010).

$$\text{Dose/kg: } D_b = D_a \times (\frac{k_b}{k_a}) \times (W_a/W_b)^{\frac{1}{3}}$$

The above formula applies to experimental animals. $Dose_a$ (D_a) is the dose of known animal (mg/per); $Dose_b$ (D_b) is the dose of unknown animal (mg/per); k_a, k_b is the body shape coefficient; W_a, W_b is the weight of animals.

And we can get the formula as follow:

$$D_{mouse} = D_{human} \times (\frac{k_{human}}{k_{mouse}}) \times (W_{human}/W_{mouse})^{\frac{1}{3}}$$

According to the SQW instruction, the dose of adult is 5.4 g/60 kg·d. And the K_{mouse} and K_{human} are 0.1057 and 0.0899 respectively, based on Experimental Methodology of Pharmacology.

So, the dosage of SQW is:

$$D_{mouse} = 90mg/kg \times (\frac{0.0899}{0.1057}) \times (60/0.02)^{\frac{1}{3}}$$

$$D_{mouse} = 1103.97mg/kg \approx 1.104g/kg \cdot d$$

D_{mouse} (1.104g/kg·d) is the SQW M, and then we get the SQW H (2.208g/kg·d) and SQW L (0.552g/kg·d) doses.

The same way we get the dose of totrodine in mice:

$$D_{mouse} = 0.0667mg/kg \times (\frac{0.0899}{0.1057}) \times (60/0.02)^{\frac{1}{3}}$$

$$D_{mouse} = 0.8178mg/kg \approx 0.82mg/kg \cdot d$$

3. Antibodies for western blot

Supplementary Table. 1 List of antibodies used for western blot

Antigen	Manufacturer	Application	Dilution
Myosin Va	Santa Cruz, Cat: sc-365986 mouse monoclonal	WB	1:1000
SLC17A9	MBL, Cat: BMP079 rabbit monoclonal	WB	1:1000
β-actin	4A Biotech, rabbit monoclonal	WB	1:1000

Reference:

Commission, C.P. (2015). *Pharmacopoeia of the People's Republic of China.* Beijing: China Medical Science Press.

Wei, W., Wu, X., and Li, Y. (2010). *Experimental Methodology of Pharmacology 4th editon.* Beijing: People's Medical Publishing House.
